# Novel germline variants identified in the inner mitochondrial membrane transporter *TIMM44* and their role in predisposition to oncocytic thyroid carcinomas

**DOI:** 10.1038/sj.bjc.6603455

**Published:** 2006-10-31

**Authors:** E Bonora, C Evangelisti, F Bonichon, G Tallini, G Romeo

**Affiliations:** 1 Unità di Genetica Medica, Policlinico Universitario S. Orsola-Malpighi, Università di Bologna, Bologna, Italy; 2 Médecine Nucléaire, Institut Bergonié, Bordeaux cedex, France; 3 Dipartimento di Anatomia Patologica, Bellaria Hospital, Università di Bologna, Bologna, Italy

**Keywords:** thyroid cancer, oncocytic cells, mitochondria, mutations

## Abstract

Familial Non-Medullary Thyroid Carcinoma (fNMTC) represents 3–7% of all thyroid tumours and is associated with some of the highest familial risks among all cancers, with an inheritance pattern compatible with an autosomal dominant model with reduced penetrance. We previously mapped a predisposing locus, *TCO* (*T*hyroid tumour with *C*ell *O*xyphilia) on chromosome 19p13.2, for a particular form of thyroid tumour characterised by cells with an abnormal proliferation of mitochondria (oxyphilic or oncocytic cells). In the present work, we report the systematic screening of 14 candidate genes mapping to the region of linkage in affected TCO members, that led us to identify two novel variants respectively in exon 9 and exon 13 of *TIMM44*, a mitochondrial inner membrane translocase for the import in the mitochondria of nuclear-encoded proteins. These variants were co-segregating with the TCO phenotype, were not present in a large group of controls and were predicted to negatively affect the protein (exon 9 change) or the transcript (exon 13 change). Functional analysis was performed *in vitro* for both changes and although no dramatic loss of function effects were identified for the mutant alleles, subtler effects might still be present that could alter Timm44 function and thus promote oncocytic tumour development. Thus we suggest that *TIMM44* should be considered for further studies in independent samples of affected individuals with TCO.

Thyroid cancer represents ∼1% of all human malignancies, but it is the most common endocrine malignancy and its incidence is increasing faster than any other malignancy in women (Malchoff and Malchoff, 2006). In addition, because thyroid nodules are frequently found with a prevalence up to 50% in areas with iodine deficiency, the assessment of a possible malignant nature of a thyroid nodule represents a serious medical problem (Schmid and Farid, 2006).

Familial Non-Medullary Thyroid Carcinoma (fNMTC) represents 3–7% of all thyroid tumours and is associated with some of the highest familial risks among all cancers, with a risk for first-degree relatives of 5/10-fold. Inheritance patterns indicate that fNMTC is an autosomal dominant trait with reduced penetrance but a multigenic inheritance could not be excluded (Lesueur *et al*, 1999).

A particular form of thyroid carcinoma is the oncocytic tumour, characterised by at least 75% of mitochondria-rich, eosinophilic cells (oxyphilic or oncocytic cells). Tumours composed of oncocytic cells may occur at a variety of sites but are particularly common among thyroid tumours of follicular cell derivation (reviewed by Tallini (1998)) and can behave aggressively possibly due to decreased competence in iodine uptake by the tumour cells and the consequent reduced responsiveness to radioiodine treatment (Tallini, 1998).

It can be difficult to differentiate benign from malignant oncocytic thyroid tumours and the overall mortality/morbidity rate of oncocytic carcinoma appears to be higher (Stojadinovic *et al*, 2001; Stojadinovic *et al*, 2002) than that of non-oncocytic papillary or follicular thyroid carcinomas even when the neoplasm is encapsulated (Ghossein *et al*, 2006).

Thyroid oncocytic cells have been associated with mitochondrial abnormalities, such as deficiencies in energy-related functions, impaired protein synthesis, and loss of electron transport components (Maximo and Sobrinho-Simoes, 2000).

Defective mitochondrial ATP synthesis has been reported in thyroid oncocytic tumours and cell lines, suggesting that reduced oxidative phosphorylation might induce mitochondrial hyperplasia and proliferation in these tumours as a compensatory mechanism (Savagner *et al*, 2001a, 2001b). We recently identified mutations in two mitochondrial DNA-encoded subunits resulting in severe loss of mitochondrial activity in a cell model of thyroid oncocytic tumours (Bonora *et al*, 2006). However, it is still not clear how nuclear and mitochondrial DNA variants influence the development of thyroid oncocytic tumours.

In collaboration with the International Consortium for the Genetics of fNMTC, we previously mapped a predisposing locus, *TCO* (*T*hyroid tumour with *C*ell *O*xyphilia, MIM#603386) to the 19p13.2 region (Canzian *et al*, 1998). The advantage of having several multigenerational pedigrees with multiple affected individuals led us to refine the TCO region on chr19 to a 1.6 Mb interval (McKay *et al*, 2004). We focused on this interval in order to identify the TCO predisposing gene. Several genes involved in mitochondrial pathways, tumour development or thyroid functions mapping to this region were analysed: *EDG5*; *MARCH2*; *LASS1*; *CCL25*; *ANGPL4*; *TIMM44*; *ELAVL1; RAB11B*; *LASS4*; *ADAMTS10*; *PIN1*; *UBL5; GRIM19;* (*NIS*). *EDG5* is involved in ceramide regulation and apoptosis; *MARCH2* is a novel membrane-bound protein with a domain similar to the cytochrome *c* binding domain. *LASS1* and *LASS4* are human homologues of the yeast longevity assurance gene *LAG1*, which regulates ceramide levels and apoptosis (Riebeling *et al*, 2003). RAB11B is a GTP-binding protein involved in trafficking and fusion of vesicles from ER to Golgi (Hales *et al*, 2001). *ADAMTS10* codes for a member of metalloproteinases. A role in carcinogenesis has been shown for *ADAMTS1*, as its overexpression reduced both tumorigenesis and metastasis *in vivo* (Kuno *et al*, 2004). *UBL5* encodes for an ubiquitin-like protein, involved in protein degradation through the proteasome pathway (Friedman *et al*, 2001). *PIN1* codes for an essential prolyl isomerase involved in the apoptotic response mediated by p53/p73 proteins (Mantovani *et al*, 2004). *ELAVL1* codes for a RNA-binding factor expressed in a wide variety of cells and involved in RNA degradation (King *et al*, 2000). *CCL25* encodes for a chemokine molecule (Soldevila *et al*, 2004). *ANGPL4* codes for a factor promoting angiogenesis and is overexpressed in several tumours (Tait and Jones, 2004). *ANGPL4* expression is regulated by several factors, such as peroxysome activating protein and hypoxia inducible factor and it is important for the regulation of metabolic processes (Ge *et al*, 2004). *TIMM44* codes for a mitochondrial inner translocase, a protein involved in the mitochondrial import of nuclear encoded proteins. The Timm44 protein is localised to the matrix and it is loosely associated to the inner membrane. Yeast Tim44 interacts with mtHsp70 during protein translocation across membranes (Bauer *et al*, 1999). Interestingly the vertebrate homologue of mtHsp70 is GRP75, a stress-induced protein (Schneider *et al*, 1994). Mitochondrial translocases such as Tim44 are highly conserved from yeast to human.

*GRIM19* codes for an effector of apoptosis, and is also part of the mitochondrial Complex I. As several new missense variants in *GRIM19* were identified in sporadic cases and in two families with multiple affected individuals presenting oncocytic tumours (Maximo *et al*, 2005), this gene was screened in a larger panel of multigenerational families such as the ones we collected.

Sodium/iodide symporter is a specific iodide transporter in thyroid follicular cells, which mediates iodide uptake across the basolateral membrane (Dohan *et al*, 2003). Several studies have reported alterations in *NIS* transcription in thyroid tumours (Saito *et al*, 1998; Lazar *et al*, 1999). As oncocytic thyroid tumours are often unresponsive to radioiodine therapy, one could hypothesise the presence of variants affecting the transcription/translation or maturation of iodide transporter in these tumours.

## MATERIALS AND METHODS

### Families and controls

The eight families with thyroid oncocytic tumours included in the study were the same as described previously (McKay *et al*, 2004). Healthy donors of Caucasian origin were included as controls. The study was approved by the relevant ethical committees, according to the rules set by the European Comunity.

### Gene characterisation

Genomic structure for each gene was obtained by BLAST comparison (http://www.ncbi.nlm.nih.gov/BLAST) of the coding mRNAs with the genomic sequence from Ensembl genome browser Build 36 (http://www.ensembl.org). The following mRNA were considered as reference sequences: NM_004230 for *EDG5*; NM_001005415 for *MARCH2*; NM_198207 for *LASS1*; NM_005624 for *CCL25*; NM_016109 for *ANGPL4*; NM_006351 for *TIMM44*; NM_001419 for *ELAVL1;* NM_004218 for *RAB11B*; NM_024552 for *LASS4*; NM_030957 for *ADAMTS10*; NM_006221 for *PIN1*; NM_024292 for *UBL5;* NM_015965 for *GRIM19;* NM_000453 for *NIS*. Exon–intron boundaries were identified and primers designed to cover exons and regulatory splice site regions using the program Primer3 (http://www.genome.wi.mit.edu/cgi-bin/primer/primer3_www.cgi). Promoter regions were determined using Promoterscan (http://zeon.well.ox.ac.uk) and PromoterInspector from the Genomatix package (www.genomatix.de).

### Mutation screening by direct sequencing

Genomic DNA was extracted from blood as previously described (IMGSAC, 1998). Polymerase chain reaction conditions were the following: 40 ng of genomic DNA, 1.5–3 mM MgCl_2_, 0.2 mM each dNTP (Roche, Indianapolis, IN, USA), 0.2 *μ*M each primer, and 0.5 U Taq Gold Polymerase (Applied Biosystems, Foster City, CA, USA) in a standard KCl buffer, final volume of 25 *μ*l. For GC-rich regions, PCR reactions were performed with the addition of 5% DMSO and 100 *μ*M 7-deaza-2′-deoxyguanosine-5′-triphosphate (Amersham Pharmacia Biotech, Little Chalfont, UK). Forty or 44 cycles depending on the primer conditions were performed with a touch-down protocol (14 cycles of 30 s at 95°C, 30 s at T1 −0.5°C/cycle, 30 s at 72°C, followed by 26–30 cycles of 30 s at 95°C, 30 s at T2, 30 s at 72°C). For GC-rich regions, each PCR step (denaturation, annealing, and elongation steps) was 1 min. Polymerase chain reaction conditions and primer sequences are available on request from the authors.

Polymerase chain reaction products were purified onto Millipore PCR clean-up plates, resuspended in H_2_O mQ, and directly sequenced on both strands using BigDye v1.1 (Applied Biosystems) according to manufacturer's instructions. Samples were loaded on ABI3730 automated sequencing machines (Applied Biosystems) and analysed using Chromas version 2.0.

### Prediction analysis of aa substitutions

Polymorphism Phenotyping (PolyPhen) (http://tux.embl-heidelberg.de/ramensky/polyphen.cgi) was used to predict the possible impact of amino acid (aa) substitutions on the protein. The program is based on sequence comparison with homologous proteins; profile scores position-specific independent counts (PSIC) are generated for the allelic variants and represent the logarithmic ratio of the likelihood of a given aa occurring at a particular site relative to the likelihood of this aa occurring at any site (background frequency). Position-specific independent counts score differences above 2 indicate a damaging effect; scores between 1.5 and 2 suggest that the variant is possibly damaging, whereas scores below 0.5 indicate that the variant is benign.

### Microsatellite analysis on chromosome 19

We examined the TCO locus in the family presenting the P308Q variant of Timm44, in the three affected sisters and the two daughters of one of them, for whom DNA was available. The DNA samples were genotyped as previously reported (Canzian *et al*, 1998). Markers were run onto ABI3730 Genetic Analyser and analysed with GeneMapper v3.5 (Applied Biosystems). Haplotype analysis was performed using Simwalk v2.91 (Sobel and Lange, 1996).

### Generation of mutated TIMM44P308Q

pcDNA3.1. plasmid containing mouse *TIMM44* cDNA (NM_011592) in frame with the V5 epitope tag was a kind gift of Dr Matsuoka (Okayama University Graduate School of Medicine and Dentistry, Okayama, Japan). Site-directed mutagenesis to insert the variant corresponding to human P308Q was performed with QuickChange XL kit (Stratagene, Amsterdam, The Netherlands) according to manufacturer's instruction, using primer forward ATCCTGAGAGTGGACCAAACCTTTGACAAGGAC and reverse GTCCTTGTCAAAGGTTTGGTCCACTCTCAGGAT. The presence of the mutation was verified by direct sequencing.

### Cell cultures

Mouse fibroblast NIH3T3, human renal HEK293 T and simian renal COS7 cells were grown in DMEM, 10% foetal bovine serum, 2 mM L-glutamine, 100 U ml^−1^ penicillin and 100 *μ*g ml^−1^ streptomycin. Cultures were grown in a humidified incubator at 37°C with 5% CO_2_.

### Co-immunoprecipitation assay

HEK293 T cells (2 × 10^5^) were transfected with 5 *μ*g pcDNA3.1V5Tim44. Wild type (wt) and mutant, using Lipofectamine (Invitrogen, Carlsbad, CA, USA) according to manufacturer's instructions. After 48 h, cells were washed once in PBS and lysed in Triton buffer (10 mM TrisHCl pH 8.0, 50 mM NaCl, 0.5%v/v Triton X-100, 10 mM PMSF and a cocktail of protease inhibitors, Roche) on ice for 15 min. Cells were collected and residual clots disrupted by passing through insulin syringe. Protein concentration was evaluated with the DC protein concentration assay Kit (Biorad, Hercules, CA, USA). Equal amount of each lysate was subjected to immunoprecipitation with 2 *μ*g anti-GRP75 goat antibody (Santa Cruz Biotechnology, CA, USA) for 2 h at 4°C in Triton buffer. Protein-G sepharose slurry (40 *μ*l) (Sigma-Aldrich, St. Louis, MO, USA) were added to each immunoprecipitation reaction for 16 h at 4°C. Immunoprecipitates were washed twice in Triton buffer for 1 min at 4°C at 8000 *g*. Proteins were eluted by heating at 95°C for 5 min in Laemli buffer and centrifugation at 8000 *g* 1 min.

Immunoprecipitates and corresponding cell lysates were separated by SDS gel electrophoresis on a 10% polyacrylamide gel, transferred onto nitrocellulose membrane (GEHealthcare, Amersham Biosciences, Little Chalfont, UK) and subjected to Western blotting with the Western Breeze kit, using mouse monoclonal anti-V5 antibody (Invitrogen) diluted 1 : 5000, according to manufacturer's instructions.

### P1 pAltermax TIMM44 exon 13-minigene generation

Polymerase chain reaction products for the genomic region surrounding exon 13 of human *TIMM44* were amplified from genomic DNA using primer forward ATGCGAATTCTACCAAGGCTGAGTGGTTCC, inserting an *Eco*RI restriction site, and primer reverse GGACCCTGAGTTGGAAGACtctagacgta, inserting a *Xba*I restriction site for cloning into the P1 pAltermax vector (a kind gift of Dr Gareth Eldvige, Wellcome Trust Centre, Oxford, UK). Polymerase chain reaction amplification was carried out using 160 ng genomic DNA from the heterozygous carrier of exon 13 variant, using the following conditions: 2.5 mM MgCl_2_, 0.5 mM dNTPs, 0.2 *μ*M primers, 5 U Pfx High Fidelity Polymerase (Invitrogen) in 50 *μ*l final volume. Thirty-five cycles were performed as follows: 95°C 2 min, 95°C 30 s, 58°C 30 s, 68°C 1 min 30 s, with a final step at 68°C for 7 min. Before purification, 0.5 U of TaqPolymerase were added to the PCR mixture in order to add protruding A, for 10 min at 72°C. The expected 913 bp long PCR product was purified onto Millipore plate and cloned into pcDNA2.1 vector using the TA cloning kit (Invitrogen), according to manufacturer's instructions. Plasmids carrying either the wt or the mutant exon 13 variant were discriminated by direct sequencing. Exon 13 products were recovered by purification with Qiagen gel extraction kit of plasmid double digestion with *Eco*RI-*Xba*I (MBI Fermentas, Hanover, MD, USA) according to standard protocol. P1 pAltermax plasmid was double-digested with *Eco*RI-*Xba*I (Fermentas), dephosphorylated with 10 U of Shrimp Alkaline Phosphatase (Fermentas) according to manufacturer's instruction, and purified by ethanol precipitation. The PCR products, either wt or mutant, were cloned into P1 pAltermax vector and plasmids sequenced.

### Splicing alteration analysis

COS7 cells were transfected with 5 *μ*g of the empty P1 plasmid or with the wt or mutant exon 13 insert, using Lipofectamine as described previously. After 48 h, total RNA was extracted using Qiagen RNeasy kit (Qiagen, GmBH, D), according to manufacturer's instructions, and subjected to RNase-free DNase I digestion (Qiagen). RNA DNase I-treated (5 *μ*g) was used for RT with Superscript III kit (Invitrogen) in a final volume of 20 *μ*l. One out of 10 of RT reaction was used for amplification with primer forward specific for pAltermax vector F AAGGCTAGAGTACTTAATACGA R and reverse specific for human *TIMM44* F CGAGAATCTGCTCGGTGCT R. Polymerase chain reaction amplification was carried out in a final volume of 25 *μ*l, 0.5 U of TaqGold Polymerase, 0.2 mM dNTPs, 0.2 *μ*M primers in a standard KCl buffer. Forty cycles were performed as follows: 95°C 15 min, 95°C 30 s 58°C 30 s 72°C 1 min. Polymerase chain reaction products were directly sequenced as described above.

## RESULTS

In order to identify the TCO predisposing gene on 19p13. 2, we have systematically screened functional candidate genes, mapping to the region of linkage, for the presence of aetiological mutations. Here, we report the analysis of 14 candidate genes mapping to the region of linkage.

Affected individuals from eight multiplex families showing linkage to the region 19p13.2 were screened for the presence of sequence variants in these genes. Once a variant was identified in one affected individual, all the other family members were tested for the presence of this change. The frequency and positions of the changes identified throughout our screening are shown in Table 1 and Supplementary Table 1. Twenty-eight changes were identified in the corresponding cDNA and by comparison with dbSNP (http://www.ncbi.nlm.nih.gov/SNP/) 12 changes were considered ‘novel’. Five changes were silent substitution (G374A, and G1274A in *TIMM44*, G509A and T971C in *ELAVL1*, C591A in *LASS1*), two changes were a G-to-T substitution in the 3′UTR of *CCL25* (G868 T) and a C-to-T substitution in *GRIM19* 5′UTR, one change was a 13 bp deletion in the 3′UTR of *MARCH2* and the others were new missense substitutions: C925A in *TIMM44* (P308Q), G1111C (R219P) in *MARCH2*, A866 T (T289 M) in *EDG5*, T1339C (V353A) in *LASS4*. The presence of all these variants was tested in the other affected individuals of the respective families and/or in the control group of healthy Caucasian individuals. From this analysis, only the two variants in *TIMM44*, C925A, and G1274A, appeared to co-segregate with the TCO phenotype in two independent families and not to be present with similar frequencies in the control group, and for this reason they were further followed up. All the new polymorphisms have been submitted to dbSNP.

### TIMM44 exon 9 variant analysis

**Figure 1:**
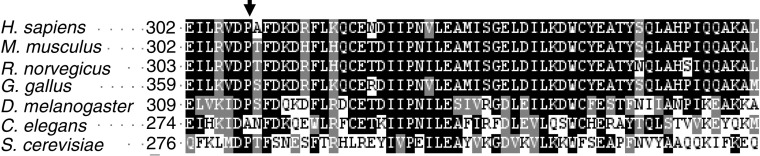
*TIMM44* conservation through species. Alignment of Timm44 proteins in different species generated using ClustalW (www.ebi.ac.uk/clustalw): the regions highly conserved are shown in black shades. The arrowhead shows the aa position corresponding to human P308.

**Figure 2:**
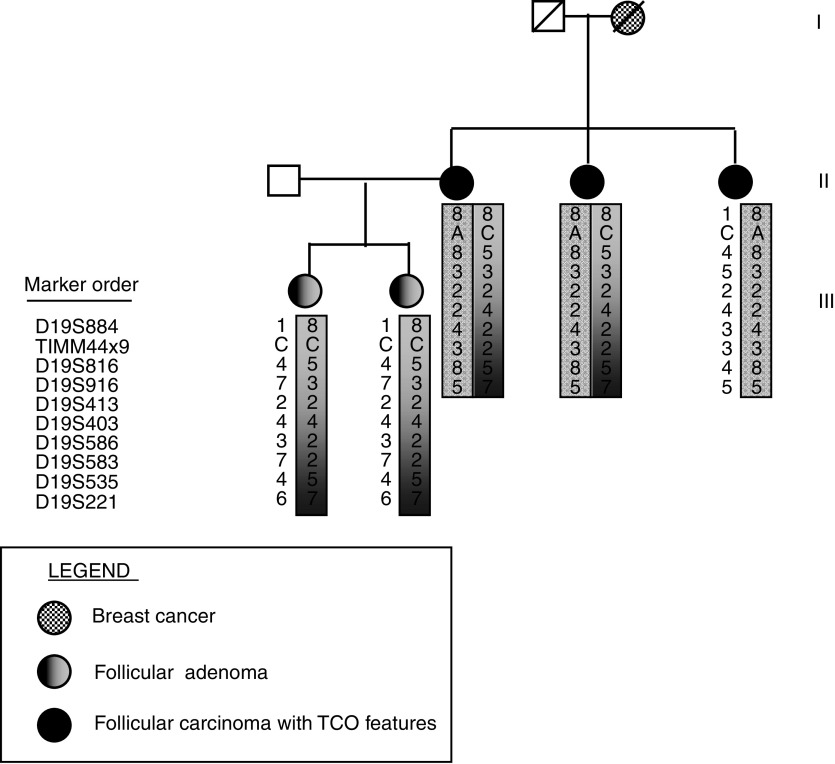
Risk haplotype on chromosome 19. Pedigree and haplotype reconstruction across 19p13.2 in the family carrying the change C925A (P308Q) in *TIMM44*. In bold it is shown the location of the exon 9 variants in *TIMM44*.

The new change in *TIMM44* C925A maps to exon 9, and corresponds to a nonconservative missense substitution, P308Q, in a highly conserved position, as indicated by clustal alignment with the orthologue proteins from different species (Figure 1). This change was found in one family and not in 125 independent controls, and it co-segregated with the TCO phenotype in the three affected sisters. Recruitment of other family members led to the analysis of two daughters of one affected member, who presented thyroid follicular adenoma but no carcinoma with oncocytic features. They also did not carry the change in exon 9 (data not shown). In addition, it was shown by the analysis with nine markers on chromosome 19 short arm, that the two daughters did not inherit the chromosome 19 risk haplotype shared by the mother and her affected sisters (Figure 2). An *in silico* analysis with PolyPhen was performed to predict the functional role of this change. P308Q is predicted to be damaging (PSIC score=2.125).

**Figure 3:**
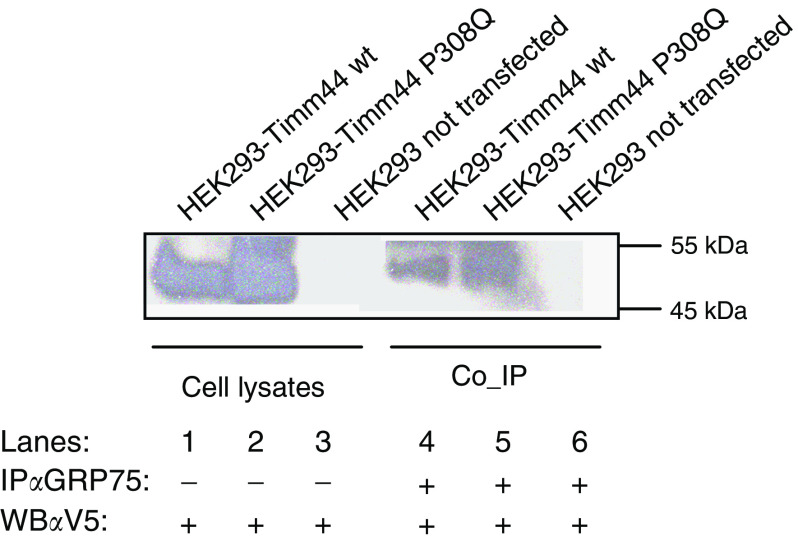
Co-immunoprecipitation experiments for Timm44 P308Q variant. Western blot analysis for V5 tag specific for recombinant Timm44 of cell lysates (lanes 1–3) and immunoprecipitates (lanes 4–6) for GRP75 of HEK293T cells transfected with the plasmid carrying either the wild type (lanes 1 and 4) or the P308Q form (lanes 2 and 5) of Timm44 and the control HEK293 T not transfected (lanes 3 and 6).

In order to test the predicted potential damaging role of P308Q change on Tim44 function, we generated a mutated mouse form, introducing the corresponding mutated aa by site-directed mutagenesis of pcDNA3.1 wild-type mouse Timm44 *in-frame* with the C-ter V5 tag. In yeast, the interaction between Tim44 and mtHsp70 is mediated by a specific region in Tim44 (aa 185–202), close to the site corresponding to human missense change P308Q (D'Silva *et al*, 2003, 2004). We thus hypothesised that in the presence of the mutated aa that inserts a nonconservative substitution from apolar to a partially charged residue, protein binding, and interaction between Timm44 and the mammalian homologue of mtHs70, GRP75 might be altered. In order to verify this hypothesis, we carried out co-immunoprecipitation experiments in mammalian cells transfected either with the normal or the mutant Tim44. Experiments were repeated twice both in mouse NIH3T3 and human HEK293 T cells with the same results in the two cell systems: immunoprecipitation for endogenous GRP75 on total cell lysates and Western blot analysis with antiV5- specific antibody for recombinant Tim44 revealed that the interaction was not abolished by the presence of the missense change in Tim44 (Figure 3 and data not shown). This result suggests that the presence of the P308Q missense change does not affect Timm44 function.

### TIMM44 exon 13 variant analysis

The second change G1274A co-segregating with the TCO phenotype and not found in 125 controls maps to exon 13 and is a silent variant. As it lies close to the 3′-splice junction, we investigated if there were putative exonic enhancer sites that might be modified by the presence of the variant. As shown by Cartegni *et al* (2006) (Liu *et al*, 2001), variants in the transcribed region of a gene may affect the binding sites (exonic enhancer) of splicing factors such as SR proteins, which promote the inclusion of that particular exon in the transcript. Alteration of these binding sites can induce the absence of that exon in the final messenger RNA, with the generation of shorter transcripts and/or truncated proteins. This phenomenon has been reported for several disorders, such as breast cancer (Liu *et al*, 2001; Fackenthal *et al*, 2002), spinal muscular atrophy (Cartegni *et al*, 2006), and hypergammaglobulinemia (Ferrari *et al*, 2001).

We used the program ESEfinder v2.0 (http://rulai.cshl.edu/tools/ESE/) to identify putative SR protein-dependent ESEs in the exon 13 wt (allele G) or mutated (allele A) and to calculate the correspondent motif scores. The A allele introduces a putative binding site for SRp40 (score=3.139; threshold=2.67) and SC35 (score=2.645; threshold=2.383) that is absent from the G allele.

**Figure 4:**
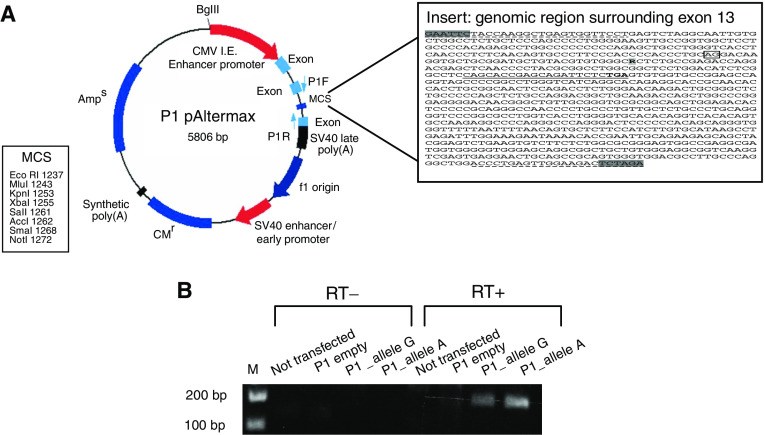
Splicing analysis for *TIMM44* exon 13 variant. (**A**) P1 pAltermax plasmid structure, showing the two synthetic exons interrupetd by an intronic sequence carrying the multiple cloning site. Abbreviation: P1-F and P1-R primers forward and reverse specific for the synthetic exons of the vector. In the box it is shown the genomic sequence including exon 13 inserted in the P1 vector. In bold it is shown the change G1276A; underlined it is shown the sequence of the reverse primer specific for human *TIMM44* encompassing the TGA stop codon; underlined and hatched it is shown the sequence of primers used for cloning, with the restriction sites shaded in grey. (**B**) RT–PCR results using the P1-F primer and the human primer specific for *TIMM44* on the cDNA from COS7 cells not transfected (lanes 1 and 5) transfected with the empty P1 vector (lanes 2 and 6), allele G-containing vector (lanes 3 and 7), allele A-containing vector (lanes 4 and 8). Lanes 1–4: no superscript in the cDNA reaction mix; lanes 5–8 superscript present in the cDNA reaction mix. The PCR products are correctly visible only in the RT+ lanes of cells transfected with the vectors carrying the two alleles (lanes 7 and 8).

To understand if the change in exon 13 might alter the splicing pattern of Tim44 transcript *in vivo*, the exon and flanking genomic regions were amplified from the heterozygous patient and the two allelic variants were independently cloned into a minigene plasmid, P1 pAlterMax. This plasmid contains two synthetic exons separated by an intronic sequence with canonical splice junctions and with a multiple cloning where to insert the fragment with a putative effect on splicing (Figure 4A).

COS7 cells were transfected either with each allele and with the empty vector. Reverse transcription–polymerase chain reaction on total RNA was performed using primers specific for the minigene only as a control of RT, for the synthetic exon (forward primer), and for the human Tim44 cDNA (reverse primer). Using the couple of primers specific for both minigene and Tim44, no signal could be detected correctly from cells transfected with the empty vector. Cells transfected with the wt or with the mutant form produced the same PCR product the size of which (150 bp) was consistent with the correct splicing between the first synthetic exon and *TIMM44* exon (Figure 4B). Resequencing confirmed the correct sequence of the PCR fragment (data not shown). No difference was identified between the wt and the two allelic variants of exon 13.

## DISCUSSION

The present work reports the systematic screening of positional and functional candidate genes predisposing to a familial form of thyroid cancer with oxyphilic cells. Familial clustering of oncocytic tumours has been reported and our group has previously mapped a predisposing locus on chromosome 19p13.2, which is associated in families with the transmission of an autosomal dominant trait with reduced penetrance (Canzian *et al*, 1998; McKay *et al*, 2004).

It has been recently pointed out by Sobrinho-Simoes *et al* (2005) that every type of thyroid neoplasm has its oncocytic counterpart; however the complex interplay between genetic and environmental factors that gives rise to this tumour phenotype is not clear. The fact that oncocytic tumours can be both multiple and familial strongly suggests that there is a germline mutation conferring an increased liability to develop this type of thyroid tumour. The gene, which could be referred to as TCO because it confers a liability to oncocytic follicular cell tumour development, is likely to be involved in at least some sporadic oncocytic tumours. In this way, the presence of several families presenting a TCO phenotype is of great help to identify the predisposing gene.

We therefore decided to screen for mutations candidate genes mapping to 19p13.2 in the families contributing to linkage to the region, with a priority for genes involved in mitochondria and/or tumour development.

No relevant variants were identified in *EDG5*; *MARCH2*; *LASS1*; *CCL25*; *ANGPL4*; *ELAVL1; RAB11B*; *LASS4*; *ADAMTS10*; *PIN1*; *UBL5*; *GRIM19; NIS*. *GRIM19* was previously analysed by another group and some missense variants were identified in sporadic and familial cases, but not in the original family where the locus on chromosome 19 was identified (Maximo *et al*, 2005). Our negative findings confirm the idea that this gene is not related to predisposition to familial forms of TCO. In accordance with our results, no mutations in *NIS* were identified in sporadic thyroid tumours in previous published screenings (Russo *et al*, 2001). However, these studies did not include familial forms of TCO, which significantly contributed to the linkage on chromosome 19p13.2, where the gene maps.

Our screening identified two interesting *TIMM44* variants in two different families. These variants co-segregated with the oncocytic carcinoma phenotype and were not present in a large number of controls. One change was a a missense variant in exon 9 and the second change a silent variant that inserted a putative exonic enhancer site in exon 13. We analysed the effects on *TIMM44* splicing, considering that in other forms of cancer, for example, breast cancer owing to *BRCA1* and *BRCA2* mutations (Liu *et al*, 2001; Fackenthal *et al*, 2002), abnormal splicing owing to the presence of variants altering exonic enhancer binding sites for different SR factors can account for a significant percentage of cases. In the present work we could not detect any splicing alteration, ruling out a functional role for the variant in exon 13 of *TIMM44*.

The analysis of the variant in exon 9, which results in the nonconservative substitution P308Q, is more intriguing. In general, coding SNPs that change the aa sequence and most likely influence function are found at a lower rate and with lower frequency than silent substitutions (Cargill *et al*, 1999). In the present case, the substitution affects the, P308 human transcript residue that is highly conserved through evolution (Figure 1), that is predicted to be damaging, and that might affect protein function. The presence of the same mutation on the risk haplotype shared by all the three affected members with oncocytic thyroid carcinoma, but not in the individuals of the same family presenting adenoma without oncocytic features, suggests that this change might be relevant for the development of oncocytic thyroid carcinoma. The role of Tim44 in mitochondrial import of nuclear-encoded proteins has been extensively studied in yeast, where it has been shown that Tim44 is present at the inner mitochondrial membrane on the matrix side and promotes the transfer of unfolded proteins passing through the inner mitochondrial channel formed by the integral membrane proteins Tim17 and Tim23 by binding to mtHsp70 (D'Silva *et al*, 2004). MtHsp70 in turn recruits Mseg1 and through ATP hydrolysis completes the passage in the matrix of mitochondrial proteins (Krimmer *et al*, 2000).

An important role for Tim44 in regulating mitochondrial functions has been recently shown by Matsuoka *et al* (2005), who demonstrated that introducing wild type Tim44 in a mouse model of diabetes reduced the production of reactive oxygen species (ROS) and decreased the neointimal proliferation of injured arteries in diabetic mice.

An excess of ROS can damage mitochondria and mtDNA, eventually contributing to promote tumour development (Eng *et al*, 2003). An enhanced ROS generation has been observed in inherited complex I deficiencies (Pitkanen and Robinson, 1996; Luo *et al*, 1997). It has been reported that transmitochondrial cytoplasmatic hybrids or cybrids (Vergani *et al*, 1995) carrying the three most common Leber's hereditary optic neuropathy pathogenic mutations in complex I subunit genes show a partial respiratory defect as well as a significant increase in ROS production (Floreani *et al*, 2005).

We recently detected in a cell model of oncocytic tumours an increased production of ROS, in presence of specific disruptive complex I and III mitochondrial DNA mutations (Bonora *et al*, 2006). These findings are compatible with poorly functioning or nonfunctional mitochondria in oncocytic neoplasms. If Tim44 has a fundamental role in protein transport and ROS production regulation, any impairment in its functions might be related to oncocytic proliferation.

We tested whether the binding to the human homologue of mtHsp70 was impaired by the P308Q variants. As shown in the results, no difference was observed suggesting that the P308Q variant does not impair Tim44. However, we cannot rule out a more subtle effects in protein transport, such as less effective transfer of the proteins from the inner import channel. Further analysis is warranted to study the role of P308Q with the development of oncocytic alterations, possibly using *Saccharomyces cerevisiae* models where the different steps of mitochondrial import have been defined in details (for a review see Krimmer *et al*, 2000).

Association studies would greatly help understanding the role of *TIMM44* in TCO and in defining, if present, a risk haplotype for the gene. It has been calculated that a sample size of 130–160 affected sib-pairs is needed to provide 80% power at the 10^−5^ significance level to detect a locus having a genotypic risk ratio of 2 and a moderate allele frequency (*P*=0.1–0.4) (Risch and Merikangas, 1996). Nevertheless, similar sample sizes are very difficult to achieve, given the rarity of the familial TCO phenotype. We also tested the presence of the variants in *TIMM44* in a panel of sporadic NMTC cases, including 41 oncocytic carcinomas, but we did not detect any variants in this set of samples (data not shown). However, mutations in different genes encoding for mitochondrial proteins might lead to the development of oncocytomas, in particular preliminary data from our group suggest that mutations in mitochondrially encoded mitochondrial proteins play a relevant role in sporadic cases (Gasparre, in preparation). Thus, a complex interplay between nuclear and mitochondrial genes can be hypothesised and we suggest that replication studies with particular attention to the presence of the variant in exon 9, in other independent samples of families affected by TCO will greatly help to understand the role of *TIMM44* in TCO predisposition and development.

## Figures and Tables

**Table 1:** Changes in cDNA of TCO candidate genes on 19p13.2

**PCR product**	**Change in cDNA**	**Type of aa change**	**dbSNP**	**Het frequency in TCO patients (%)**	**Het frequency in controls (%)**
*TIMM44*x4	G344A	Silent	–	12.5	NA
***TIMM44*x9**	**C925A**	**P308Q**	–	**12.5**	**0**
***TIMM44*x13**	**G1274A**	**Silent**	–	**12.5**	**0**
*TIMM44*x13	C1307T	Silent	rs1157923	12.5	NA
*ELAVL1*x3	G509A	Silent	–	12.5	NA
*ELAVL1*x5	T971C	Silent	–	12.5	NA
*ELAVL1*x5	A4024G	3′UTR	rs12983784	37.5	NA
*CCL25*x3	A303G	H101R	rs2032887	62.5	NA
*CCL25*x3	C312T	T104M	rs2113089	12.5	NA
*CCL25*x4	G379A	Silent	rs2303164	37.5	29.7
*CCL25*x5	G675A	3′UTR	rs2287936	25.0	NA
*CCL25*x5	G868T	3′UTR	–	12.5	NA
*ADAMTS10*x4	C646G	T134S	rs7255721	37.5	NA
*ADAMTS10*x26	T3548G	H1101Q	rs7252299	90.0	NA
*ANGPTL4*x7	G1362A	Silent	rs11672433	12.5	NA
*LASS1*x3	C591A	Silent	–	8.0	NA
*LASS4*x9	C997T	Silent	rs1127912	12.5	NA
*LASS4*x9	T1006C	Silent	rs36247	37.5	NA
*LASS4*x12	T1339C	V353A	rs17160348	12.0	NA
*LASS4*x12	G1376A	A366T	rs36259	88.0	NA
*RAB11B*x5	C558T	Silent	rs2230876	57.0	NA
*MARCH2*x1	C20T	5′UTR	rs12150986	42.0	NA
*MARCH2*x2	A615G	T54A	rs11882865	67.0	NA
*MARCH2*x5	G1111C	R219P	rs34099346	42.9	22.5
*MARCH2*x5	del(CTTCCACTTCAAC) bp1267-1280	3′UTR	–	13.0	3.0
*GRIM19*x1	C182T	5′UTR	–	25.0	NA
*EDG5*x2	A919C	Silent	rs2116942	14.2	NA
*EDG5*x2	A866T	T289M	–	14.2	NA

aa=amino acid; cDNA=complementary; NA=not available; PCR=polymerase chain reaction; TCO=thyroid tumor with cell oxyphilia.
