# Supplementary material for: Novel germline variants identified in the inner mitochondrial membrane transporter TIMM44 and their role in predisposition to oncocytic thyroid carcinomas
Source: Br J Cancer. 2006 Oct 31;95(11):1529–36. doi: 10.1038/sj.bjc.6603455 (PMC2360750; doi:10.1038/sj.bjc.6603455)
Supplement: Supplementary Table 1 [file 95-6603455x1.doc]

Supplementary table 1: SNPs identified in intronic and regulatory regions in TCO candidate genes.

| Variant | Contig position (bp) | Type of change | dbSNP | Het frequency in TCO patients (%) | Het frequency in controls  (%) |
| --- | --- | --- | --- | --- | --- |
| *TIMM44* Prom | 613121 NT_077812 | T→G | rs17551975 | 12.5 | na |
| *TIMM44* Prom | 612480 NT_077812 | C→T | rs12985279 | 12.5 | na |
| *TIMM44* int8 | 601679 NT_077812 | C→T | -- | 12.5 | na |
| *TIMM44* int11 | 596953 NT_077812 | A→G | -- | 12.5 | na |
| *ELAVL1*int1 | 930940 NT_077812 | C→T | rs17855413 | 12.5 | na |
| *ELAVL1*int3 | 636676 NT_077812 | G→A | rs1077340 | 12.5 | na |
| *ELAVL1*int4 | 636428 NT_077812 | C→T | rs3826740 | 12.5 | na |
| *ADAMTS10* int4 | 1273869 NT_077812 | A→G | rs7260282 | 38.0 | na |
| *ADAMTS10* int16 | 1258912 NT_077812 | C→T | rs3923268 | 20.0 | na |
| *LASS1* int5 | 10252661 NT_011295 | C→T | rs2075762 | 17.0 | na |
| *LASS4* int5 | 924655 NT_077812 | Δ(gcggggcggg) | -- | 50.0 | 74.0 |
| *LASS4* int7 | 925178 NT_077812 | A→T | rs28334 | 38.0 | na |
| *LASS4* int9 | 925942 NT_077812 | T→C | rs36247 | 38.0 | na |
| *EDG5* Prom | 45979 AC_011511 | C→A | -- | 36.0 | 36.0 |
| *MARCH2* int1 | 1086932 NT_077812 | C→T | -- | 11.0 | na |
| *MARCH2* int1 | 1087304 NT_077812 | T→C | rs12150986 | 35.7 | na |
| *MARCH2* int4 | 1099761 NT_077812 | G→A | rs2303180 | 60.0 | na |
| *ANGPTL4* int1 | 1032995 NT_077812 | C→G | rs4076317 | 42.9 | na |
| *UBL5* int5 | 1203616 NT_011295 | C→G | rs2287839 | 12.0 | na |
| *RAB11B* int5 | 1203616 NT_011295 | C→G | rs2287839 | 14.0 | na |
